# Serving the contraceptive needs of HIV-positive women in Africa: the need for a health systems focus

**DOI:** 10.1186/1748-5908-10-S1-A22

**Published:** 2015-08-20

**Authors:** Theresa Hoke

**Affiliations:** 1 Health Services Research Division, FHI 360, Durham, NC 27707, USA

## Research goal

Global policy supports service integration to ensure that the contraceptive needs of women using HIV/AIDS services are met. We conducted an intervention study in 5 public sector health facilities in South Africa to test a strategy for serving the reproductive health (RH) needs of postpartum PMTCT clients. We conducted another study in 8 public sector health facilities in Uganda to test an intervention for increasing dual method use (condom + another contraceptive) among clients receiving antiretroviral therapy. Both studies failed to demonstrate increases in contraceptive prevalence. We examined process data to understand why RH-HIV service integration was not successful when tested in routine practice.

## Methods

To complement the two intervention trials, we conducted process evaluations to evaluate intervention implementation. Using a tracking tool, we systematically documented how intervention components were implemented, noting how actual implementation compared to the original design. The tool documented problems encountered during implementation. It provided space for the study team to make recommendations on adjustments to be made for future implementation.

## Results

Process data from both studies revealed problems with intervention implementation that were rooted in health system constraints. These included inadequate human resource management, weaknesses in commodity management, poor coordination to support referrals, and inadequate reporting mechanisms. The process evaluation revealed that for RH-HIV service integration to succeed in routine service delivery contexts, attention must be directed toward reinforcing the health system components undergirding service delivery. These two research experiences highlighted the importance of process evaluation to know why interventions fail.

Results on the implementation process and the influence of context where interventions are introduced should be systematically reported so that trials results can be interpreted accurately and health service managers can confront systemic problems that limit health service innovations.

Funding for both studies was provided by the US Agency for International Development.

